# Genome-wide association mapping unravels the genetic control of seed germination and vigor in *Brassica napus*

**DOI:** 10.3389/fpls.2015.00221

**Published:** 2015-04-09

**Authors:** Sarah V. Hatzig, Matthias Frisch, Frank Breuer, Nathalie Nesi, Sylvie Ducournau, Marie-Helene Wagner, Gunhild Leckband, Amine Abbadi, Rod J. Snowdon

**Affiliations:** ^1^Department of Plant Breeding, Justus Liebig UniversityGiessen, Germany; ^2^Department of Biometry and Population Genetics, Justus Liebig UniversityGiessen, Germany; ^3^KWS SAAT AGEinbeck, Germany; ^4^Institute for Genetics, Environment and Plant Protection, INRALe Rheu, France; ^5^Groupe d'Etude et de contrôle des Variétés Et des SemencesBeaucouzé, France; ^6^NPZ Innovation GmbHHoltsee, Germany

**Keywords:** seedling, emergence, high-throughput phenotyping, GWAS, rapeseed, canola

## Abstract

Rapid and uniform seed germination is a crucial prerequisite for crop establishment and high yield levels in crop production. A disclosure of genetic factors contributing to adequate seed vigor would help to further increase yield potential and stability. Here we carried out a genome-wide association study in order to define genomic regions influencing seed germination and early seedling growth in oilseed rape (*Brassica napus* L.). A population of 248 genetically diverse winter-type *B*. *napus* accessions was genotyped with the *Brassica* 60k SNP Illumina genotyping array. Automated high-throughput *in vitro* phenotyping provided extensive data for multiple traits related to germination and early vigor, such as germination speed, absolute germination rate and radicle elongation. The data obtained indicate that seed germination and radicle growth are strongly environmentally dependent, but could nevertheless be substantially improved by genomic-based breeding. Conditions during seed production and storage were shown to have a profound effect on seed vigor, and a variable manifestation of seed dormancy appears to contribute to differences in germination performance in *B. napus*. Several promising positional and functional candidate genes could be identified within the genomic regions associated with germination speed, absolute germination rate, radicle growth and thousand seed weight. These include *B. napus* orthologs of the *Arabidopsis thaliana* genes *SNOWY COTYLEDON 1* (*SCO1*), *ARABIDOPSIS TWO-COMPONENT RESPONSE REGULATOR* (*ARR4*), and *ARGINYL-t-RNA PROTEIN TRANSFERASE 1* (*ATE1*), which have been shown previously to play a role in seed germination and seedling growth in *A. thaliana*.

## Introduction

Selection bottlenecks have a large impact on the diversity available for breeders to sustain selection gains for important traits, particularly in crop species like modern oilseed rape (*Brassica napus* L.) with relatively small gene pools. One approach to overcome this problem is introgression of untapped germplasm in order to broaden the genetic basis of breeding materials (Friedt and Snowdon, 2010). A main focus in genetics and breeding of oilseed rape has been on analysis and improvement of general breeding targets, such as abiotic and biotic stress resistances (e.g., Obermeier et al., 2013; Hatzig et al., 2014) or flowering time optimization (Schiessl et al., 2014). Enhancement of such traits can contribute directly to an increase and stabilization of yield levels. On the other hand, less attention has been given to elucidation of the genetic control of seed germination and seedling vigor, although these are fundamental processes shaping field emergence (Matthews and Khajeh Hosseini, 2006; Wagner et al., 2011; Matthews et al., 2012) and yield performance (Larsen et al., 1998).

Germination is a major component of seed vigor, which is defined as the sum of those properties of the seed that determine the potential level of activity and performance of seed lots with acceptable germination under a wide range of environments (Perry, 1978). Although seed germination is influenced by both genetic as well as environmental factors, it is still an open question whether seeds performing well under optimal conditions also have advantages under stress conditions. However, different studies dealing with drought (Bettey et al., 2000), salt or cold stress (Foolad et al., 1999) strengthen such correlations.

Germination *per se* commences with the uptake of water by the quiescent dry seed, interruption of dormancy and the subsequent elongation of the embryonic axis (Bewley, 1997). Water uptake during germination is a triphasic process while per definition germination is completed upon entry into the third phase (Bewley, 1997). Initially, water uptake is driven by physical swelling of the seed when hydrophilic molecular groups form hydration shells. There is evidence that environmental influences already affect germination during this initial phase, in which water uptake takes place independently from metabolism. Factors like temperature or the presence of specific solutes are able to retard or accelerate seed swelling (Leopold, 1983). Subsequent to physical swelling, metabolic processes are initiated and germination becomes irreversible by water deprivation. This second phase is also described as the plateau phase (Bewley, 1997), as increases in fresh weight due to physical swelling are already completed, while active growth takes place due to cell differentiation and elongation after the initiation of underlying biochemical processes. Per definition germination stops upon entry into the third phase, in which growth becomes visible and the primary root breaks through the testa (Bewley, 1997).

For breeding of vigorous plant cultivars with fast and uniform field emergence, it is crucial to understand the genetic factors that contribute to an adequate germination performance and seedling growth. As seed germination is a very complex trait controlled at transcriptional, translational and metabolic level (Rajjou et al., 2012), it is difficult to identify the contributing genetic factors by conventional genetic or physiological analyses. A preferential method for identification of genomic regions associated with complex quantitative traits is genome-wide association mapping, in which marker-trait associations are calculated across a broad set of diverse germplasm in order to define chromosome regions harboring promising genes.

The high resolution of genome-wide association studies, arising from the incorporation of numerous ancient meioses in a set of unrelated plant individuals, has become a popular method for disclosure of genomic polymorphisms affecting quantitative traits. However, a common drawback of GWAS is that population structure can shift allele frequencies within a diversity panel. This can lead to the detection of false-positive marker-trait associations (Korte and Farlow, 2013) or a non-detection of rare and/or region-specific alleles (Nordborg and Weigel, 2008). In such a case statistical methods have to be applied which correct for population stratification.

In this study we used genome-wide association studies for the investigation of quantitative trait loci (QTL) linked to germination performance and early vigor in winter oilseed rape (*Brassica napus* L.). An automated high throughput phenotyping platform (Demilly et al., 2014) was used to assay diverse traits, related to the three different phases of seed germination and post-germination radicle growth, in a large set of diverse winter rapeseed lines genotyped with genome-wide single-nucleotide polymorphism (SNP) markers. Genome-wide association analyses using these data sets enabled identification of highly promising candidate genes and markers for breeding towards improved germination in rapeseed. Phenotyping of seed lots produced in different environments helped to evaluate the effects of germination-independent factors, such as seed ripening and storage, on germination performance.

## Materials and methods

### Seed material and phenotyping

A total of 248 genetically diverse, winter-type *B. napus* inbred lines were used in this study, including 216 winter oilseed rape (WOSR), 20 winter fodder (WF) and 10 exotic lines (Table S1). Seeds of all inbred lines were produced by controlled self-pollination in two distinct environments during the growing seasons of 2010/2011 (SL 2011) in Le Rheu, France and 2011/2012 (SL 2012) in Asendorf, Germany. The genotype panel comprises lines derived from modern oilseed rape cultivars with low seed erucic acid and glucosinolate content, old rapeseed varieties with high seed erucic acid and high glucosinolate content (++ quality), fodder rapes, kale vegetable forms, and resynthesized *B. napus* derived from interspecific hybridizations between its two diploid progenitors, *Brassica rapa* and *Brassica oleracea*.

Monitoring of seed imbibition, germination, and early radicle growth was conducted under *in vitro* conditions using the automated phenotyping platform of the variety control office of the French national seed testing agency (Station Nationale d'Essais de Semences, Groupe d'Etude et de contrôle des Variétés et des Semences—GEVES, Angers, France). The phenotyping platform is described in detail by Ducournau et al. (2004, 2005) and Wagner et al. (2011). Image acquisition, image analysis and data analysis methods are described in detail by Demilly et al. (2014). The following parameters were determined: Volume increase within first 8 h (VI; in %), imbibition speed during first 4 h after initiation of imbibition (IS; in mm^3^/h), total germination rate within 72 h after initiation of imbibition (GR72; in %), first germination time (FG; in h), mean germination time (MGT; in h), radicle elongation speed (ES; in mm/h), time to reach 50% of germination (T50; in h) and germination rate within 36 h after initiation of imbibition (GR36; in %). Additionally, thousand seed weight (TSW; in g) was measured before germination monitoring. Correlations were calculated applying the Pearson's product-moment correlation.

### Pre-processing of marker data

Genomic DNA was extracted from young leaf materials for all genotypes of the diversity panel (*n* = 248). Genotyping was performed with the *Brassica* 60k Illumina® Infinium consortium SNP array (Edwards et al., 2013), according to standard procedures of the manufacturer and using the same cluster file for SNP calling. For physical localization of SNP markers, flanking sequences were blasted onto the *Brassica napus* Darmor-*bzh* reference genome sequence assembly (version 4.1), recently published by Chalhoub et al. (2014). SNPs were blasted using the following criteria: minimum overlap of 50 bp length, minimum identity of 95%, no sequence gaps. SNPs which failed across the entire genotype collection, as well as all locus non-specific SNPs, for which more than 1 BLAST hit on the *B. napus* sequence was found, were excluded from further analyses. As heterozygous SNPs cannot be distinguished from multi-locus hemi-SNPs or false calls, heterozygous calls were treated as missing values. For visualization of population structure and calculation of genome-wide associations, SNP markers with more than 20% missing calls across the panel were excluded. Furthermore, all individuals which had more than 20% missing calls across the genotype data were excluded. In order to incorporate rare alleles with potential effects on germination performance, only markers with a minor allele frequency ≤ 0.025 were excluded from analysis. After pre-processing, 218 individuals and 22,169 SNP markers remained for further analyses.

### Population structure analysis

Visualization of genetic relatedness was performed using the statistical software R studio version 0.98.501 and the integrated R package *SelectionTools* (http://www.uni-giessen.de/population-genetics/downloads). Genetic distances were calculated applying the euclidean modified Rodger's distance method according to Wright (1978). In order to visualize genetic relatedness among all genotypes, principal component analysis (PCA) was carried out regarding the first four components. Correction for population stratification was performed by a mixed-model approach including principal component covariates. According to Price et al. (2010) this is the method of choice for correction of population structure, family structure and cryptic relatedness. Genotypes were assigned to specific clusters by k-means clustering (MacQueen, 1967).

Optimal number of clusters was estimated by calculating the within-cluster sum of squares (WSS), varying cluster number k from 1 to 15, and subsequently defined as 4. Linkage disequilibrium (LD) was calculated for each chromosome individually, quoting coefficients of determination (r^2^) for all locus pairs localized on the same chromosome. Inter-chromosomal genome-wide LD decay was calculated for trait-associated markers that exhibited unexpected patterns of local LD. Locally paired scatterplot smoothing in R was employed for graphical representation of LD curves with a span of 0.1.

For characterization of genomic kinship, genome-wide allele identity by descent was computed for the whole diversity panel as well as separately for the four identified subpopulation clusters, using the R package GenABEL (Aulchenko et al., 2007). Additionally, to determine the proposition of genetic differentiation explained by differentiation among the subclusters, overall F_ST_ values were calculated using the software Genepop version 4.2.2 (Rousset, 2008).

### Genome-wide association analysis

Narrow-sense heritability for each trait was calculated separately for each seed lot, again using the R package GenABEL. Genome-wide associations were calculated with GenABEL. Adjustment for stratification was performed by mixed-model approximation combined with PC adjustment (Price et al., 2010; Svishcheva et al., 2012). For PC adjustment the first 4 components were taken into account.

False non-discovery rate was calculated with the R package *fdrtool* based on p-value statistics. Only associations exceeding the predetermined cutoff values were taken into account. For the integration of markers with weak associations to the phenotypic traits, all SNPs with a −log_10_(*p*-value) > 3 were considered as significant. Marker-trait associations were regarded as reliable when they could be confirmed in a second seed lot with −log_10_(*p*-value) > 2.5. To define regions of interest for selection of potential candidate genes, local LD decay was first calculated within the flanking regions up to 1000 kbp on either side of the associated markers. All genes anchored between the two markers next to an associated marker were taken into account for the disclosure of candidates. Furthermore, when flanking markers were in strong LD (*r*^2^ > 0.4) with an associated marker, regions were defined as LD blocks and the whole LD block was taken into account for candidate gene disclosure. Genes within LD blocks containing trait-associated markers were characterized with the tool Blast2GO (Conesa et al., 2005) using default settings, and candidates were selected based on their gene ontology (GO) terms. For differentiation between coding and non-coding regions of selected candidate genes the Genscan web server (http://genes.mit.edu/GENSCAN.html) was used. The plant pathway database MetNet (http://www.metnetonline.org) was used to determine common pathway memberships among the identified candidate genes.

Marker haplotypes were defined for each associated region and genotypes were bulked in regard to their associated-marker haplotypes. For T50 and ES boxplots were generated showing the phenotypic distribution of 4 and 6 classes, respectively, representing the cumulative phenotypes from haplotypes with favorable effects on the trait performance.

### Analysis of chlorophyll content

For chlorophyll analyses, two groups of 10 extreme genotypes were selected with consistently high and low values, respectively, for ES (radicle elongation speed) in both seed lots. Seeds from SL2011 were used for chlorophyll content determination. Around 60 seeds of each genotype were germinated in Jacobsen vessels on circular filter papers soaked with H_2_O dest. Continuous water supply was ensured by wicks connecting the filter papers with 100 mL H_2_O dest at the bottom of each vessel. Germination was carried out in a climate chamber with the following conditions: 20°C, 16 h light, 8 h dark. Chlorophyll extraction was performed after 5 d. The cotyledons of eight seedlings per genotype were pooled and treated as one biological replicate. In total 3 biological replicates were sampled per genotype. Fresh weights of all samples were measured. Subsequently all samples were homogenized (Ultra-Thurrax T25, IKA, Staufen / Germany) in 15 mL Falcon tubes filled with 5 mL dimethyl sulfoxide (DMSO, >99.8% GC). The samples were incubated at room temperature for 24 h in the dark. For chlorophyll quantification, 1 mL of the supernatant was collected and filled into a micro cuvette (1.6 mL) for spectrometric measurement. The absorbance of the sample solutions was measured after blank calibration (1 mL DMSO) with a SmartSpec Plus spectrophotometer (Bio-Rad Inc., Hercules, CA, USA) at 665 nm and 649 nm wavelength. Chlorophyll a and chlorophyll b concentrations were calculated according to the equations of Wellburn (1994). Significant differences were calculated with Student's *t*-test.

## Results

### Phenotypic distribution, genetic diversity and linkage disequilibrium

Phenotypic values for all traits showed a similar mean trait performance and phenotypic variability in the two seed lots SL2011 and SL2012 (Table 1). However, only small to medium correlations (0.24 < *r* < 0.63) were calculated between the two seed lots. Strongest correlations were found between MGT, T50 and GR36, with I r I > 0.9, and between VI and IS (I r I > 0.7). Correlations with 0.3 ≤ I r I ≤ 0.5 were observed between FG and the traits VI, MGT, ES, T50 and GR36, as well as between VI and MGT, T50 and GR36 (Table 2).

**Table 1:** **Phenotypic variation for different traits associated with seed germination performance in two seeds lots of a *Brassica napus* diversity panel (n=248) produced in Le Rheu, France in 2011 (SL2011) and Asendorf, Germany in 2012 (SL2012), respectively: Minimum (Min), maximum (Max), standard deviation (SD) and correlation between seed lots (r)**.

**Trait**	**Unit**	**SL2011**				**SL2012**				
		**Min**	**Max**	**Mean**	***SD***	**Min**	**Max**	**Mean**	***SD***	***r***
TSW	g	3.07	6.70	4.65	0.53	3.12	6.24	4.92	0.54	0.63^***^
VI	%	8.12	40.37	22.33	5.08	7.91	46.43	24.14	6.57	0.44^***^
IS	mm^3^/h	0.02	0.35	0.18	0.05	0.00	0.40	0.17	0.07	0.24^***^
FG	h	12.00	30.00	21.85	3.81	12.00	28.00	17.43	3.35	0.26^***^
MGT	h	27.62	48.75	36.29	3.41	27.17	46.16	35.88	3.22	0.56^***^
T50	h	26.14	62.00	35.14	3.88	27.08	45.75	35.20	3.22	0.57^***^
GR36	%	7.00	93.33	55.89	18.62	10.00	87.00	54.78	15.73	0.58^***^
GR72	%	44.00	100.00	96.96	5.53	75.00	100.00	94.68	4.00	0.44^***^
ES	mm/h	0.51	1.24	0.81	0.12	0.61	1.23	0.88	0.12	0.57^***^

*Abbreviations of traits are explained in Materials and Methods*.

Significances were calculated at a level of

*** *p < 0.001*.

**Table 2:** **Correlation coefficients (r) for different trait pairs associated with seed germination performance measured in two seed lots (SL2011 and SL2012) from an oilseed rape diversity panel (*n* = 248)**.

	**TSW**	**VI**	**IS**	**FG**	**MGT**	**T50**	**GR36**	**GR72**
VI	−0.04							
IS	0.21^***^	0.74^***^						
FG	−0.08	−0.30^***^	−0.10^*^					
MGT	0.10^*^	−0.41^***^	−0.19^***^	0.46^***^				
T50	0.15^**^	−0.38^***^	−0.18^***^	0.36^***^	0.94^***^			
GR36	−0.15^***^	0.39^***^	0.19^***^	−0.37^***^	−0.95^***^	−0.94^***^		
GR72	−0.08	−0.14^**^	−0.08	0.06	−0.15^***^	−0.36^***^	0.27^***^	
ES	0.17^***^	0.25^***^	0.17^***^	−0.36^***^	−0.29^***^	−0.18^***^	0.19^***^	−0.23^***^

*Abbreviations of traits are explained in Materials and Methods*.

Significances were calculated at levels of

* p < 0.05,

** p < 0.01 and

*** *p < 0.001*.

**Figure 1:**
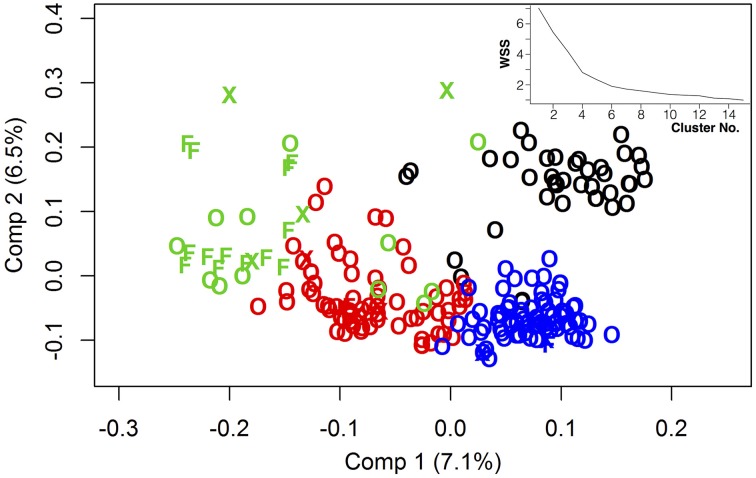
**Genetic relatedness of 218 genetically diverse winter-rapeseed lines shown in a principal component analysis regarding the first 2 components**. Different clusters were represented by different colors. Winter oilseed rape lines were shown as “O,” winter fodder lines as “F” and others as “X.” Top right: Within-cluster sum of squares depending on the number of clusters applied for k-means clustering.

**Figure 2:**
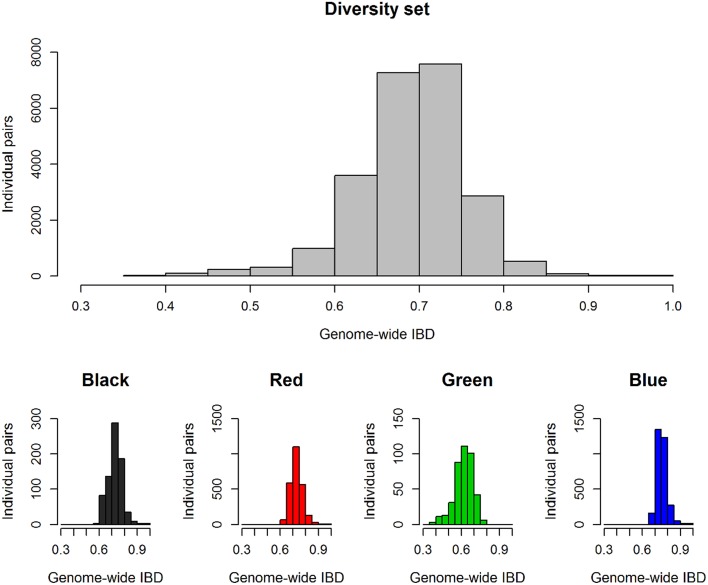
**Genetic relationship between all individual pairs within the total diversity set (*n* = 218) and within the single clusters from k-means clustering**. Histograms show the frequency of individual pairs depending on their kinship coefficients IBD (identity by descent) which score allelic identity by descent.

With regard to genetic relatedness, the first four PCA components explained 20.98% of the total genetic variance, while the first two components contributed 7.1% (Comp 1) and 6.5% (Comp 2), respectively (Figure 1). Almost all fodder rape genotypes (13 out of 16) were assigned to the same cluster (herein referred to as cluster “green”). Taking k-means clusters into account, an overall F_ST_ value of 0.1448 was observed over the whole diversity set, indicating a medium genetic differentiation. Regarding the population pairs, lowest genetic differentiation was found between clusters “red” and “blue” (F_ST_ = 0.10), while strongest differentiation was calculated between clusters “green” and “blue” (F*_ST_* = 0.21). For all genotype pairs within the diversity set, a mean identity by descent (IBD) of 0.69 was calculated, while values of 0.72, 0.73, 0.62, and 0.76 were found within clusters “black,” “red,” “green,” and “blue,” respectively (Figure 2). The largest cluster “blue” comprised 79 genotypes and showed the narrowest distribution, whereas the smallest cluster “green” comprised only 29 genotypes but showed the widest distribution of IBD values.

**Figure 3:**
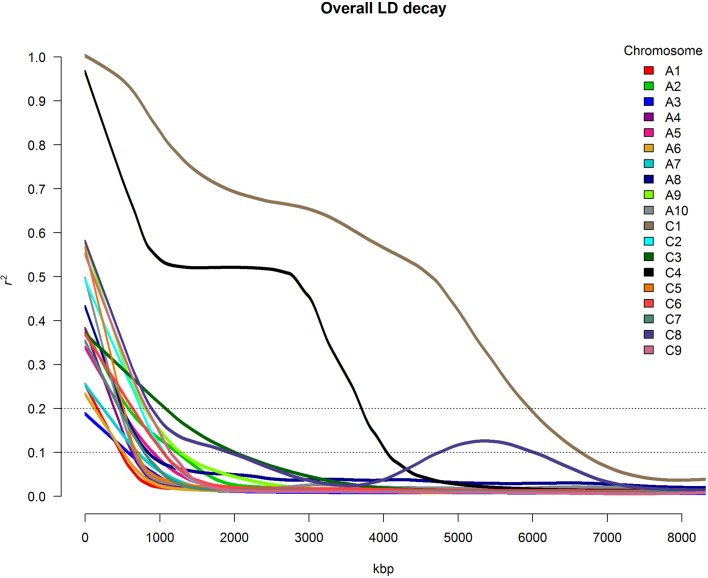
**Overall chromosome-wide decay of linkage disequilibrium (LD), shown as smoothed *r*^2^-values for all marker pairs on each chromosome depending on the distance between marker pairs**.

For all chromosomes except C1, C3, C4, and C8, LD decayed to *r*^2^ = 0.1 at a distance between marker pairs ranging from 480 kbp (A1) to 1283 kbp (A9) (Figure 3). For chromosomes C3 and C8, r^2^ did not drop to 0.1 until the distance between marker pairs reached 2014 kbp and 1939 kbp, respectively. For chromosome C8, strongly conserved LD was observed for marker distances between 4500 and 6000 kbp. Particularly strong patterns of LD were observed on chromosomes C1 and C4, with *r*^2^-values above 0.1 for up to 6651 kbp and 4048 kbp, respectively. The very slow average LD decay on chromosome C4 was mainly determined by the presence of a large conserved LD block localized between 15,429 and 20,449 kbp, whereas conserved LD between 17,787 and 26,635 kbp caused the extremely slow LD decay on chromosome C1.

Narrow-sense heritability differed considerably among traits and for some traits also between seed lots (Table 3). The strongest differences between the 2011 and 2012 seed lots were observed for the trait T50, with relatively low heritability observed within the 2011 seed lot and comparatively high heritability within the 2012 seed low. The traits GR36, ES and TSW showed a medium to high heritability with *h*^2^ > 0.5 in all cases. For VI and FG a low heritability was calculated with *h*^2^ > 0.3. For GR72 the heritability approached zero for both seed lots. Marker-trait associations that could be confirmed in both seed lots were detected for T50, GR36, GR72, ES, VI, FG, and TSW (Table 3). The observed F_ST_ value revealed a low to medium genetic differentiation within the diversity set (Figure 2), with subdivision into clusters explaining 14.5% of the total genetic variation. Calculation of the inflation factor λ by genomic control (Devlin and Roeder, 1999; data not shown) confirmed that population structure was predominant. Hence, correction for stratification was performed by mixed-model approach including principal component covariates. Subdivision into clusters caused an increase of IBD values within all clusters except for cluster “green,” which mainly comprises fodder rapes and exotic accessions.

**Table 3:** **Intervals of linkage disequilibrium (LD) containing significant marker-trait associations for different traits related to germination performance in seeds of an oilseed rape diversity panel produced in Le Rheu, France in 2011 (SL2011) and Asendorf, Germany in 2012 (SL2012), respectively: Chromosome (Chr), Chromosomal position in base pairs [LD interval (bp)], narrow-sense heritability (h^2^), additive allelic effect (Add. eff.), logarithmic scaled *P*-value (−log_10_(p))**.

**Trait**	**Chr**	**LD interval (bp)**	**SL2011**			**SL2012**		
			***h*^2^**	**Add. eff**.	**−log_10_(p)**	***h*^2^**	**Add. eff**.	**−log_10_(p)**
TSW	A1	2,094,614 – 2,132,814	0.84	−0.23	3.28	0.76	−0.23	2.65
	A5	18,641,259 – 18,796,724		−0.28	2.53		−0.35	3.11
	A6	22,333,133 – 22,588,108		0.26	5.32		0.19	2.95
	C6	21,335,770 – 21,666,697		−0.13	2.66		−0.15	3.07
VI	C7	41,147,197 – 41,318,742	0.23	1.69	3.43	0.18	2.05	2.69
FG	C7	35,089,636 – 35,279,703	0.21	0.91	2.60	0.29	1.11	4.07
T50	A9	32,206,450 – 32,410,348	0.34	2.76	4.80	0.86	1.98	3.06
	C6	31,736,330 – 32,133,403		1.10	2.74		1.23	3.85
	C8	36,326,419 – 36,384,818		1.46	2.60		1.41	3.02
GR36	C8	36,326,419 – 3,638,4818	0.50	−8.36	3.47	0.77	−6.64	2.76
GR72	A3	4,478,594 – 4,515,865	0.07	−2.01	3.16	0.00	−1.78	4.28
		7,082,702 – 7,098,892		−1.96	2.64		−1.82	3.69
		16,074,818 – 16,173,009		−2.44	3.43		−2.01	4.22
	A4	4,102,377 – 4,144,135		−2.37	3.41		−2.01	4.04
		4,260,229 – 4,302,660		−2.21	3.13		−1.99	4.33
		11,328,102 – 11,529,657		−2.17	3.03		−1.79	3.59
	A9	32,206,450 – 3,241,0348		−3.00	3.00		−2.86	3.16
	C6	31,736,330 – 32,133,354		−1.59	3.10		−1.06	2.58
	C9	42,980,135 – 43,015,797		−1.89	2.70		−1.91	4.68
ES	A9	6,512,728 – 7,122,368	0.62	−0.04	2.75	0.72	−0.05	4.22
	A10	11,631,861 – 11,677,068		−0.07	3.00		−0.08	3.14
		16,085,669 – 16,164,217		0.05	3.15		0.05	3.00
	C2	12,742,547 – 14,316,723		0.04	2.63		0.04	3.37
	C6	25,600,351 – 25,671,165		0.04	2.68		0.06	4.73

*Abbreviations of traits are explained in Materials and Methods*.

### Genome-wide association analysis

The chromosomal regions delineated by haplotype blocks in strong LD (*r*^2^ > 0.4) with trait-associated markers harbored a total of 681 genes (Table S2). Several genes ascribed to seed germination, seed dormancy and seed and embryo development were disclosed, associated with T50, GR72, ES, FG, and TSW (Table 4). Further investigation showed that none of the candidate genes listed in Table 4 was known to be involved in a mutual pathway. Identical chromosome regions were associated with T50 and GR72, hence the same candidate genes were assumed on chromosomes A9 and C6. The SNP marker Bn-A06-p23586019, associated with TSW, is localized within the non-coding region of the candidate gene *BnaA06g34100D*. Very strong local LD values (*r*^2^ > 0.8) were found between markers Bn-A04-p4074166, Bn-A04-p4217227 and Bn-A04-p10196289, associated with GR72 on chromosome A4, whereas LD with the other surrounding markers was very low (0 < *r*^2^ < 0.06). On the other hand, these markers showed strong extra-chromosomal LD to markers Bn-A03-p5072729 and Bn-A03-p16990947 on chromosome A03, both of which also showed associations with GR72. Although this might be due to co-selection of two epistatically interacting gene loci, this may also suggest a spurious allocation of markers Bn-A04-p4074166, Bn-A04-p4217227 and Bn-A04-p10196289 to the wrong chromosome, hence they were excluded from further analysis.

**Table 4:** **List of candidate genes ascribed to seed germination, dormancy and seed development: Chromosome (Chr), Name of the candidate gene (Candidate gene) and absolute chromosomal position in base pairs [Position (bp)]**.

**Trait**	**Chr**	**Candidate gene**	**Position (bp)**
TSW	A6	BnaA06g34100D	22,551,497 – 22,558,918
		BnaA06g33970D	22,490,437 – 22,491,055
		BnaA06g33830D	22,421,795 – 22,424,461
		BnaA06g33880D	22,446,550 – 22,447,630
	C6	BnaC06g19160D	21,499,780 – 21,506,256
FG	C7	BnaC07g31020D	35,273,679 – 35,275,807
T50	A9	BnaA09g48160D	32,313,276 – 3,231,4748
	C6	BnaC06g31150D	31,787,778 – 31,792,013
GR72	A9	BnaA09g48160D	32,313,276 – 32,314,748
	C6	BnaC06g31150D	31,787,778 – 31,792,013
	C9	BnaC09g40630D	42,994,450 – 42,995,360
		BnaC09g40640D	42,997,059 – 42,998,540
ES	A9	BnaA09g12770D	6,802,082 – 6,804,959
		BnaA09g12820D	6,856,835 – 6,858,406
		BnaA09g12800D	6,834,031 – 6,835,814
		BnaA09g13040D	7,099,948 – 7,100,930
	A10	BnaA10g24850D	16,143,505 – 16,146,463
		BnaA10g24780D	16,125,049 – 16,127,616

*Abbreviations of traits are explained in Materials and Methods*.

**Figure 4:**
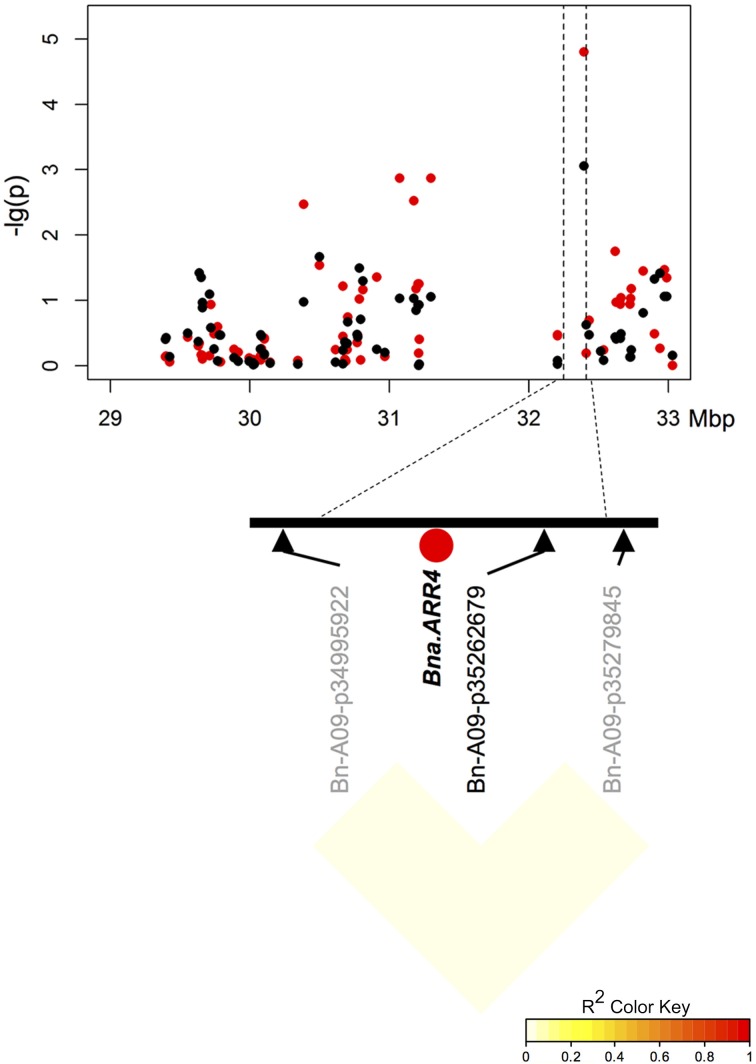
**Manhattan plot describing marker-trait associations for the trait ES within the chromosomal region around candidate *BnaA09g48160D (Bna.ARR4)* on chromosome A9, and correlations between surrounding markers**. Red dots represent associations calculated for SL2011. Black dots represent associations for SL2012.

**Figure 5:**
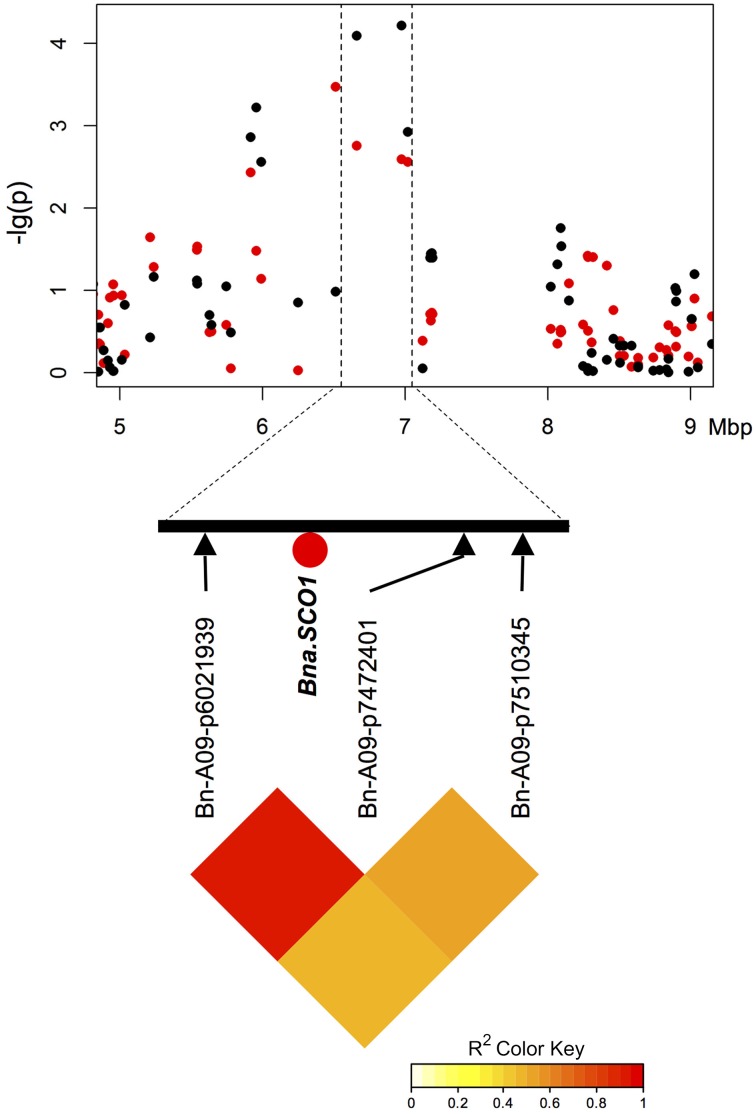
**Manhattan plot describing marker-trait associations for the trait T50 within the chromosomal region around candidate *BnaA09g12770D (Bna.SCO1)* on chromosome A9, and correlations between the associated marker and both flanking markers**. Red dots represent associations calculated for SL2011. Black dots represent associations for SL2012.

**Figure 6:**
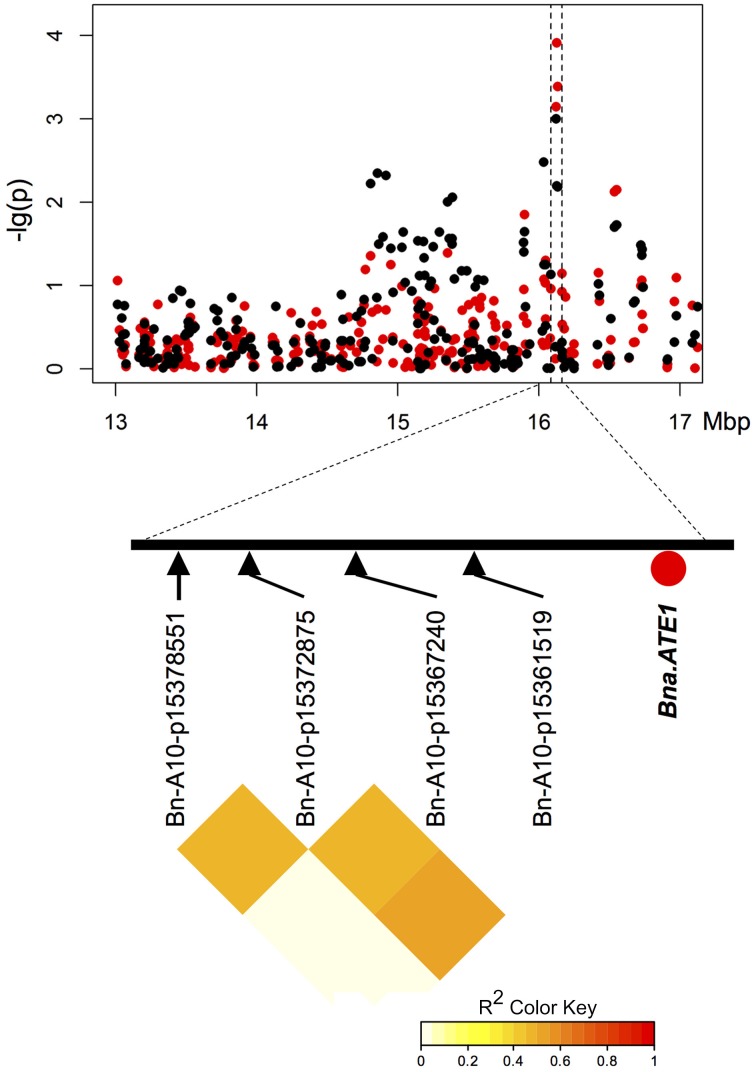
**Manhattan plot describing marker-trait associations for the trait ES for the chromosomal region around candidate *BnaA10g24850D (Bna.ATE1)* on chromosome A10, and correlations between markers of the linkage group harboring *BnaA10g24850D***. Red dots represent associations calculated for SL2011. Black dots represent associations for SL2012.

Two of three particularly promising candidate genes were associated with blocks of strong LD containing QTL for different traits on chromosomes A9 (two independent QTL) and A10. The first of these three, *BnaA09g48160D*, is located on chromosome A9 only 80.789 kbp from marker Bn-A09-p35262679, which shows significant associations to T50 (−log_10_(p) = 4.8 and 3.1 and GR72 (−log_10_(p) = 3.2 and 3.0), but exhibits almost no LD to its nearest flanking markers (Tables 3, 4, Figure 4). *BnaA09g48160D* encodes for an ortholog of *A. thaliana* gene *ARABIDOPSIS TWO-COMPONENT RESPONSE REGULATOR* (*ARR4*). In another independent association peak between 6.51 and 7.12 Mbp on chromosome A9 we localized the gene *BnaA09g12770D*, between two adjacent markers with very strong LD that both show significant associations to ES (Tables 3, 4, Figure 5). The SNP marker Bn-A09-p6021939 (associated with −log_10_(p) = 4.1 and 2.8 to ES) is located 141.051 kbp from *BnaA09g12770D*, which codes for an ortholog of the *A. thaliana* gene *SNOWY COTYLEDON* 1 (*SCO1*). Another significant association peak for ES was localized on chromosome A10 (Tables 3, 4). This region harbors an LD block containing four markers (Bn-A10-p15378551, Bn-A10-p15372875, Bn-A10-p15367240, and Bn-A10-p15361519) the latter of which lies directly adjacent (9.701 kbp) to the gene *BnaA10g24850D* (Figure 6). *BnaA10g24850D* is an ortholog of another *A. thaliana* gene implicated in germination and early development, *ARGINYL-t-RNA PROTEIN TRANSFERASE 1* (*ATE1*).

**Figure 7:**
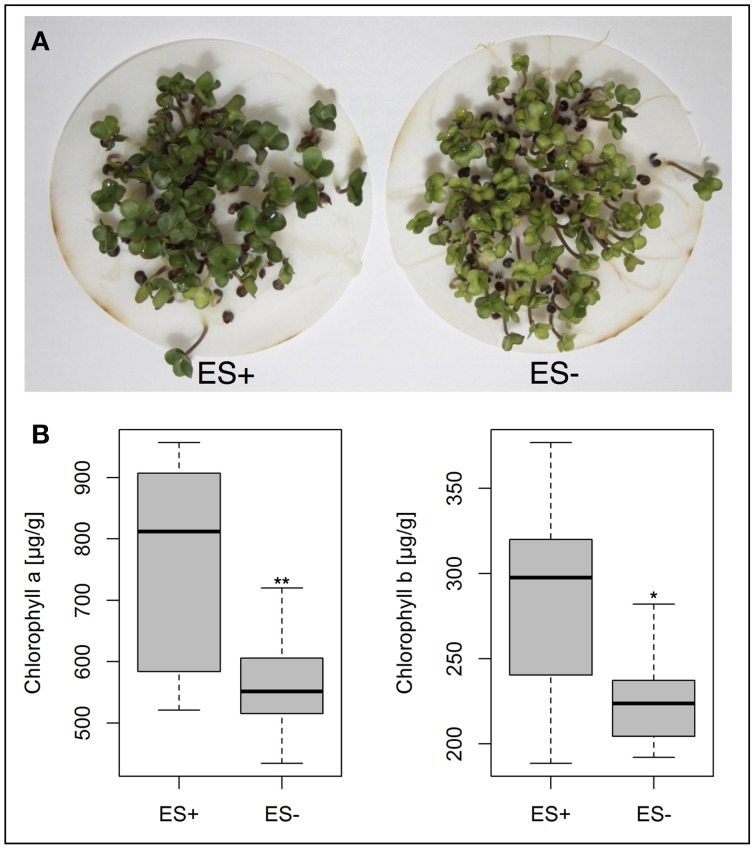
**(A)** Phenotypic variation for cotyledon chlorophyll pigmentation in *B. napus* genotypes showing high (ES+) and low (ES−) elongation radicle rates. **(B)** Box plots representing chlorophyll a and chlorophyll b concentrations of 10 genotypes each, showing high and low elongation radicle rates. Significant differences were calculated with student's *t*-test at *p* < 0.05^*^ and *p* < 0.01^**^.

Pigment concentrations were measured in the cotyledons of 5d old seedlings from the two genotype bulks, featuring consistently low and high ES values, respectively. Mean concentrations of chlorophyll a and b were found to be significantly lower in genotypes with poor performance in ES (Figure 7).

**Figure 8:**
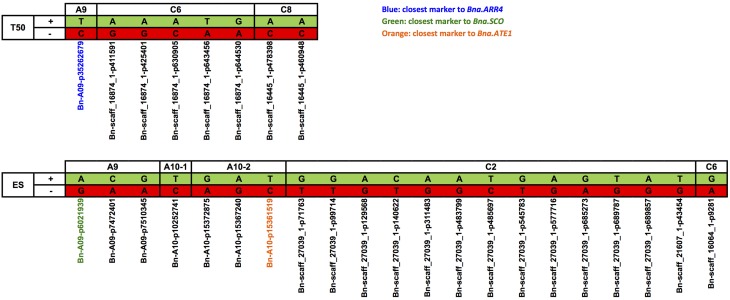
**Loci-specific alleles of single SNP markers and different haplotypes from LD-blocks harboring various SNP markers associated with T50 and ES**. Favorable alleles (+) were marked in green, alleles with negative effect on trait performance (−) were shown in red. Corresponding marker names were listed below. T50, Time to reach 50% of germination; ES, Elongation speed of the radicle.

**Figure 9:**
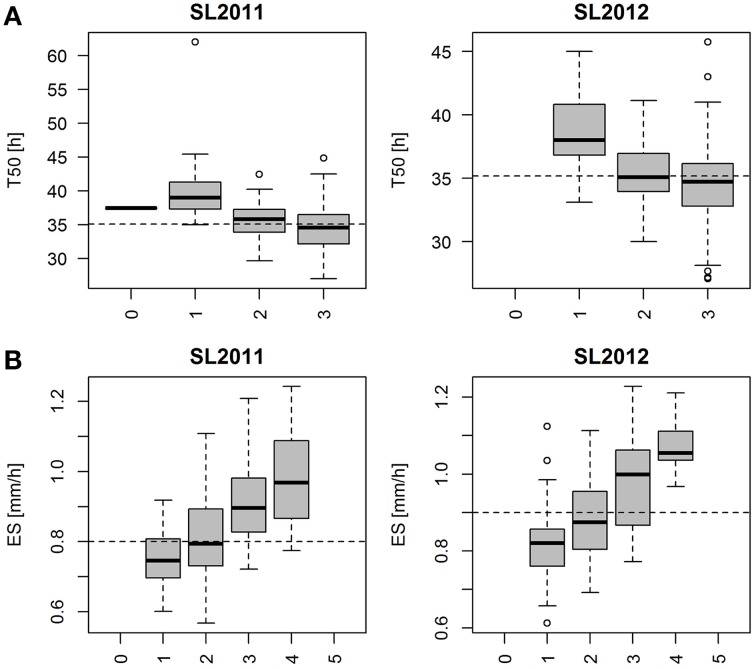
**(A)** Boxplot representing the phenotypic distribution for T50 (Time to reach 50% of germination) in two different seed lots, produced in 2011 (SL2011) and 2012 (SL2012). Each whisker represents a bulk of genotypes exhibiting no favorable haplotype (0), one favorable haplotype (1), two favorable haplotypes (2) and three favorable haplotypes (3) regarding the three associated regions on chromosome A9, C6 and C8. Population means were indicated as dashed lines. **(B)** Boxplot representing the phenotypic distribution for ES (Elongation speed of the radicle) in two different seed lots, produced in 2011 (SL2011) and 2012 (SL2012). Each whisker represents a bulk of genotypes exhibiting no favorable haplotype (0), one favorable haplotype (1), two favorable haplotypes (2) three favorable haplotypes (3), four favorable haplotypes (4) and five favorable haplotypes (5) regarding the five disclosed associated regions on chromosome A9, A6, A10, C2, and C6. Population means were indicated as dashed lines.

Marker haplotypes for all disclosed regions associated with T50 and ES are displayed in Figure 8, which compares the most frequent locus-specific haplotypes with favorable and undesirable effects on trait performance. Because a threshold of *r*^2^ > 0.4 was chosen for LD block definition, a smaller fraction of recombinant haplotypes also occurred. A list of all observed haplotypes and their frequencies is provided in Table S3. With increasing number of favorable alleles, performance is shown to be improved for both T50 and ES (Figure 9). For T50 an increase in the number of undesirable haplotypes was associated with a decrease in the number of individuals per bulk (Table S3). While the main fraction of lines (*n*=133) exhibited the favorable haplotype at all three loci, a combination of all three unfavorable alleles was observed in only one line. For ES, neither a combination of all favorable, nor all undesirable haplotypes occurred, with most individuals (*n* = 105) exhibiting 2 favorable alleles (Table S3).

## Discussion

### The key elements of seed germination

Seed germination has a decisive influence on homogeneous field emergence and successful seedling establishment and is therefore a basic target for the development of vigorous crop plants with stable yield performance. According to Finch-Savage et al. (2010), germination speed is a major key element of vigorous seeds, along with a rapid initial downward and upward seedling growth.

While traits like seed imbibition speed (IS) and seed volume increase (VI) can be used for characterization of initial water uptake within the first hours after soaking, mean germination time (MGT), time to reach 50% of germination (T50), germination rate within 36 h (GR36) and first germination (FG) provide insight into the speed of the total germination process until the radicle breaks through the testa. Elongation speed of the radicle (ES) is not part of the germination process *per se*, but is an important component of pre-emergence seedling growth after entry into the third phase of water uptake (Bewley, 1997). Although seed vigor is constituted by both germination speed and pre-emergence seedling growth, these two criteria are presumed to contribute independently to seed vigor performance (Dutt and Geneve, 2007). This assumption is strengthened by the very low or absent phenotypic correlations we observed between ES and germination speed parameters (FG, MGT, T50, GR36) in the *B. napus* diversity panel. Interestingly, we found no evidence for a decisive influence of seed weight (TSW) on germination performance, absolute germination rate or radicle growth, with only extremely low correlations being observed to these parameters. This is in accordance to Bettey et al. (2000), who attributed no influence of seed weight to mean germination time or absolute germination in *Brassica oleracea*. On the other hand, Hanumaiah and Andrews (1973) described a positive relationship between seed size and seedling growth for *B. rapa* and *B. oleracea*. According to our observations TSW should be regarded as a seed yield parameter in oilseed rape rather than a contributing factor for germination performance.

Weak correlations between germination speed (T50, MGT, GR36, and FG) and seed imbibition in terms of VI and IS indicate that the velocity of total germination is impacted only to a limited extent by initial water uptake. In turn, induction of the biochemical processes contributing to embryo expansion and leading to visible germination seem to be mainly independent from imbibition speed. Apparently other factors induce the release of germination, and in this context it is worthwhile to consider seed dormancy. According to Finch-Savage and Leubner-Metzger (2006), seed dormancy is an innate seed property that defines the environmental conditions that must be met before the seed can germinate. With respect to the diverse regulation levels, dormancy can be divided into different dormancy types (Baskin and Baskin, 2004). For members of the *Brassicaceae* it is assumed that mainly physiological dormancy is predominant (Müller et al., 2006). Our results indicate that differences in germination speed in *B. napus* are not solely dependent on water availability or the presence of favorable conditions for the induction of biochemical actions contributing to seed germination. In fact, the observed variation in germination speed might be caused by differences in the manifestation of seed dormancy. However, because the *in vitro* germination assay we applied implements optimal and controlled germination conditions, two explanations are possible: On the one hand, the diversity panel we investigated appears to exhibit differences in genetically determined responsiveness to dormancy release factors. On the other hand, the observed differences in germination performance also appear to be strongly determined by the conditions predominant during and after seed production. The latter hypothesis is strengthened by the observation of only moderate correlations between the two seed lots (Table 1). As both seed lots were tested for germination under constant laboratory conditions, the observed differences in performance between the two seed lots must reflect genotype by environment interactions during seed ripening in the field and/or during the subsequent seed storage. One possible mechanism could be epigenetic imprinting during seed development due to stress conditions in the maternal environment.

### Exploring the genetic basis of seed germination and seedling growth

Against the background of a strong environmental dependence of seed germination and early seedling growth, breeders are interested in the fixed fraction of variables influencing seed vigor performance. Although the physiology of seed germination and dormancy is well studied and extensively described in literature (e.g., reviewed in Bewley, 1997; Finch-Savage and Leubner-Metzger, 2006; Finkelstein et al., 2008; Rajjou et al., 2012), there is still a lack of knowledge about individual genes involved in these complex traits, particularly in crop species. Both germination and dormancy underlie polygenic control that can only be elucidated by quantitative genetics approaches.

To estimate how much genotypic variation contributes to the observed phenotypic variation we calculated narrow-sense heritability (h^2^), which captures only that part of genetic variation caused by additive genetic values (Visscher et al., 2008). Narrow-sense heritability can only be calculated based on the detected associations, so that undetected associations may repress *h*^2^-values. The observed high *h*^2^-values for TSW agree with findings from earlier studies (Basunanda et al., 2010; Fan et al., 2010; Khan et al., 2013; Xing et al., 2014). In contrast, very low *h*^2^-values for GR72 suggested that the absolute germination rate is almost completely dependent on the environmental state and can hardly be influenced by selection. Nevertheless, identical associations for T50 and GR72 on chromosomes A9 and C6 indicate that both germination speed and absolute germination rate seem to be controlled by the same genetic factors (see Table 3), so that selection for T50 may also lead to improvement of GR72.

The candidate gene disclosure strategy we applied accounted for trait associations that were detectable in both of the investigated seed lots. The use of local LD to delineate regions of interest around significantly associated SNP markers proved a valuable method for defining potential candidate genes. The very strong LD on some C-sub-genome chromosomes corresponds to findings in other *B. napus* gene pools and reflects extreme selection for specific seed quality traits (Li et al., 2014; Qian et al., 2014). Strong LD conservation can complicate candidate gene identification from association peaks, hence it is important to consider local LD when defining QTL confidence intervals for candidate gene selection. The validity of the trait associations we detected appears to be confirmed by the identification of comparatively small regions of strong LD that harbor highly interesting genes involved in processes closely related to seed germination and seedling development.

Several genes ascribed to seed germination, dormancy, seed development and radicle emergence were identified within the associated regions (Table 4). For example, the *A. thaliana* gene *ARR4 is* a cytokinin-dependant antagonistic regulator of transcription activator *ABI5*, which is directly involved in the ABA mediated regulation of seed germination (Wang et al., 2011). The presence of a *Bn.ARR4* homolog in a region of LD with an association peak for T50 and GR72 corresponds to the expected role of ABA-mediated dormancy and germination release. In *A. thaliana* the gene *SCO1* was found to encode a plastidic translation elongation factor G, which is reported to be essential for eoplast to chloroplast transition during early germination (Ruppel and Hangarter, 2007). Furthermore, *A. thaliana sco*1 mutations also cause a chlorophyll deficiency resulting in pale-green to white coloring of seedling cotyledons, and the seedling chlorophyll deficiency of *sco1* mutants is coupled with a delayed seed germination and development (Albrecht et al., 2006). Accordingly, *B. napus* genotypes with extreme segregation for ES also showed significant differences in seedling chlorophyll content. Associations of *Bna.SCO1* haplotypes to ES, together with the corresponding chlorophyll phenotypes, support a causal role for this gene in the speed of early radicle growth in *B. napus*. Mutations in *ATE* genes encoding for arginyl-t-rna protein transferases have also been shown to cause reductions in *A. thaliana* seedling root growth (Holman et al., 2009). Allelic differences in *Bn.ATE1* within LD haplotypes associated with ES are hence also strong positional and functional candidates for control of radicle growth during and after germination.

Genotypic classification for T50 and ES revealed that both traits can potentially be improved by pyramiding of favorable alleles (Figures 9A,B). For both traits the absence among the diversity panel of individuals with three (T50) or five (ES) unfavorable haplotypes indicates that strong natural and artificial selection has occurred in cultivated forms of *B. napus* against individuals with very poor germination and vigor. Consequently, effects of loci associated with these traits might be underestimated because specific undesirable allele combinations are absent, thus restricting the extent of phenotypic variability within the diversity set. For ES we also observed a lack of favorable haplotype combinations, indicating that early seedling growth could be substantially improved beyond the phenotypic diversity in the present study.

## Conclusions

Large-scale automated phenotyping revealed a broad phenotypic variability in germination performance across a *B. napus* diversity panel. As expected, narrow-sense heritability revealed that germination performance is influenced by both genetic as well as environmental factors. As the latter were constant during *in vitro* phenotyping, the observed differences in germination performance between two different seed lots could be attributed to the conditions predominant during seed production and storage. No relationship of germination traits to seed weight could be detected. Instead, the results indicate that genetic determinants for the manifestation of seed dormancy may be a decisive factor influencing inheritance of germination performance in *B. napus*. In a genome-wide association analysis several promising genes, including *Bna.SCO1, Bna.ARR4*, and *Bna.ATE1*, were found within regions showing LD with QTL for seed germination and vigor. Combination of positive alleles for the loci we identified should facilitate a decisive increase in germination performance and early seedling growth in oilseed rape, improving prospects for breeding of these complex, poorly selectable traits. As such the study provides a basis for the establishment of genomic selection tools for improved seed germination and seed vigor in rapeseed.

## Author contributions

SH and RS designed the study and interpreted the results. RS, NN, AA, and GL developed the diversity panel and generated seed lots for the phenotypic analysis. NN, AA, GL, and FB performed field phenotyping and analyzed the field phenotype data, SD and MW performed the automated germination phenotyping, and FB generated the genome-wide SNP data. SH and MF performed the GWAS analysis. SH and RS wrote the manuscript and all authors corrected and approved the final version.

### Conflict of interest statement

The authors declare that the research was conducted in the absence of any commercial or financial relationships that could be construed as a potential conflict of interest.

## Abbreviations

ABA: Abscisic acid
*ARR4*: *A*. *thaliana* gene *two-component response regulator ARR4 (*AT1G10470.1)
*ATE1*: *A*. *thaliana* gene *Arginine-tRNA Protein Transferase 1* (AT5G05700.1)
*Bna.ARR4*: *B*. *napus* ortholog of *A*. *thaliana* two-component response regulator *ARR4 (*AT1G10470.1)
*Bna.ATE1*: *B*. *napus* ortholog of *A. thaliana* gene *ATE1*, encoding for *Arginine-tRNA Protein Transferase 1* (AT5G05700.1)
*Bna.SCO1*: *B*. *napus* ortholog of *A*. *thaliana* gene *snowy cotyledon 1* (AT1G62750.1)
DMSO: Dimethyl sulfoxide
ES: Elongation speed of the radicle (mm/h)
FG: First germination (h)
F_ST_: Fixation index
GO: Gene onthology
GR36: Germination rate within 36 h (%)
GR72: Germination rate within 72 h (%)
GWAS: Genome-wide association study
IBD: Identity by descent
IS: Imbibition speed during first 4 h after initiation of imbibition (mm^3^/h)
LD: Linkage disequilibrium
MGT: Mean germination time (h)
PC: Principal component
PCA: Principal component analysis
*SCO1*: *A*. *thaliana* gene snowy cotyledon 1 (AT1G62750.1)
SL2011: Seed lot produced in 2011 in LeRheu, France
SL2012: Seed lot produced in 2012 in Asendorf, Germany
SNP: Single nucleotide polymorphism
T50: Time necessary to reach 50% of germination (h)
TSW: Thousand seed weight (g)
VI: Volume increase within first 8 h (%).
